# Sense of Coherence and Health-Related Quality of Life in Patients With Brain Metastases

**DOI:** 10.3389/fpsyg.2020.01516

**Published:** 2020-07-02

**Authors:** Xian Qiu, Nan Zhang, Si-Jian Pan, Peng Zhao, Bei-Wen Wu

**Affiliations:** ^1^School of Nursing, Shanghai Jiao Tong University, Shanghai, China; ^2^Department of Neurosurgery, Ruijin Hospital, School of Medicine, Shanghai Jiao Tong University, Shanghai, China; ^3^Research Center of Brain and Cognitive Neuroscience, Liaoning Normal University, Dalian, China; ^4^Gamma Knife Center, Shandong Provincial Hospital, Jinan, China; ^5^Department of Nursing, Ruijin Hospital, School of Medicine, Shanghai Jiao Tong University, Shanghai, China

**Keywords:** sense of coherence, health-related quality of life, brain metastases, awareness of illness, religious belief

## Abstract

With improvements in treatments for primary tumor and brain metastases (BM), the life expectancy of patients with advanced cancers is increasing; thus, helping patients with BM maintain quality of life is becoming increasingly important. Sense of coherence (SOC) has been found to be closely related to health-related quality of life (HRQoL) in patients with chronic diseases, however, this relationship has not been validated in patients with BM. This study first examined the relationship between SOC and HRQoL in patients with BM, and further identified factors associated with SOC in these patients. Patients with BM reported lower scores for most of the functioning subscales and for the general rating of quality of life, and higher scores for most of the symptom subscales, compared with a normative sample. SOC was significantly correlated with most aspects of HRQoL in patients with BM. Further, SOC in the patients was associated with awareness of the disease, possession of religious belief, and type of primary cancer. These results validate the close relationship between SOC and HRQoL in patients with BM, and indicate that SOC is associated with awareness of illness and religious belief.

## Introduction

Metastatic brain tumors occur when primary tumor cells from other parts of the body metastasize to the brain. It is estimated that 20–40% of patients with cancer will develop brain metastases (BM), and the incidence of BM is 4–5 times more common than that of primary brain tumors (Kalkanis and Linskey, 2010). The reported number of patients with BM is still increasing (Verhaak et al., 2020), which may partly be attributed to the fact that many chemotherapeutic agents cannot cross the blood-brain barrier (Nayak et al., 2012). Although the prognosis for patients with BM is poor, their life expectancy is increasing due to improvements in systemic management of primary tumor and BM. Therefore, clinicians are focusing on how to help these patients maintain good health-related quality of life (HRQoL) (Verhaak et al., 2020).

The occurrence of metastases may induce a high level of psychological distress in cancer patients. Some cancer survivors show successful psychosocial adjustment to the illness (Aderhold et al., 2019). Such adjustment can be closely related to sense of coherence (SOC), which is associated with reduced depressive symptoms in cancer patients (Aderhold et al., 2019). SOC is defined as “a global orientation that expresses the extent to which one has a pervasive, enduring though dynamic feeling of confidence that (i) the stimuli deriving from one’s internal and external environments in the course of living are structured, predictable, and explicable; (ii) the resources to meet the demands posed by these stimuli are available; and (iii) these demands are challenges, worthy of investment and engagement” (Chaiklin, 1989). These three aspects, respectively, correspond to the three components of SOC: comprehensibility, manageability, and meaningfulness (Antonovsky, 1993).

A few studies have indicated that in patients with chronic illnesses, SOC is positively associated with quality of life (Kristofferzon et al., 2011). A meta-analysis which included studies of cancer patients, provided empirical evidence that high SOC has a protective influence on perceived health (Winger et al., 2016). A study of patients with prostate cancer showed that a stronger SOC is associated with a higher perceived quality of life (Jacobsen et al., 2002). However, no significant relationship was found in another study between SOC and quality of life among patients with cancer, perhaps because of the small sample size of the study (Persson and Hallberg, 1995). Currently, few studies have examined the relationship between SOC and HRQoL in patients with BM.

Research suggests that in adulthood, SOC remains relatively stable, and even after radical life events, only temporary fluctuations occur. It has been shown that SOC is unaffected by age, gender, education, and place of residence (Nowicki et al., 2019). Other research has provided evidence that neither race nor education affect SOC among patients with cancer and chronic diseases (Bruscia et al., 2008). No changes in SOC was observed from 1 to 3 years after cancer diagnosis (Lindblad et al., 2016). Nevertheless, exploration of the factors associated with SOC is worthwhile, given that it is closely related to quality of life. A study indicated that cancer patients who rely on spiritual and religious beliefs to cope with their illness are more likely to use an active coping style, in which they accept their illness and try to address it in a positive and meaningful way (Weaver and Flannelly, 2004). It has been found that better academic performance compared with peers was associated with stronger SOC (Chu et al., 2016). As such, it appears that psychological factors, such as the possession of religious belief or the awareness of the malignant degree of disease, could be related to SOC in patients with advanced cancer.

Currently, studies are lacking of the association between HRQoL and SOC; nor have investigations considered the factors related to SOC in patients with BM. Accordingly, the first aim of this study was to investigate the relationship between SOC and HRQoL in patients with BM. Such knowledge would contribute to the growing body of research highlighting the close association between HRQoL and SOC in patients with chronic diseases. Further, we investigated the relationship between sociodemographic/psychological/disease-related characteristics and the level of SOC in patients with BM.

## Materials and Methods

### Patients

This cross-sectional study was conducted at the Department of Functional Neurosurgery of Ruijin Hospital and the Gamma Knife Treatment Center of Shandong Provincial Hospital from January 2017 to July 2019. The inclusion criteria were (a) 18 years of age or older, (b) a diagnosis of BM, and (c) physically able to participate. Patients were excluded from the study if they exhibited disturbed consciousness or if they were diagnosed with a psychiatric disorder, as indicated by their clinical records. The patients were enrolled during appointments either after consultation or before hospitalization. Each patient recruited was fully informed of the purpose of the study and gave their written consent to participate. Sociodemographic information and treatment histories were recorded. In addition, the patients were asked to complete questionnaires measuring SOC and HRQoL at the clinic or during the intake process for hospitalization. The patients were instructed to complete the questionnaires based on their experiences of cancer, rather than previous experiences of psychological disorders, other chronic illnesses, or previous trauma. In total, 170 patients consented to participate in the study, all of whom answered the questionnaires before gamma knife treatment. The 162 patients (95.3%) who completed the questionnaires formed the sample. This study was approved by Ruijin Hospital Ethics Committee (2017; clinical ethics approval no. 28).

### Instruments

#### Quality of Life Questionnaire Core-30

The European Organization for Research and Treatment of Cancer Quality of Life Questionnaire Core-30 Version 3.0 (QLQ-C30) is a self-rated questionnaire that assesses multiple dimensions of quality of life among cancer patients. It is composed of multi-item functional scales (physical, role, cognitive, emotional, and social functioning), multi-item scales, or single-item measures of common symptoms reported by patients with cancer patients (fatigue, pain, nausea and vomiting, dyspnea, insomnia, appetite loss, constipation, and diarrhea), financial difficulties, and two questions assessing global quality of life. An example item from the functioning subscale is “Do you have trouble taking a long walk?” and an example from the symptom subscales is “Have you had pain during the past week?” The raw score of each subscale/item in the QLQ-C30 is linearly transformed into a score ranging from 0 to 100. Higher scores on functional or global quality of life scales indicate better functioning or quality of life, while higher scores on symptom scales indicate the presence of more severe symptoms (Aaronson et al., 1993). The total score of the QLQ-C30 is calculated from the mean of 13 of the 15 scales (the Global Quality of Life scale and the Financial Impact scale are not included).

#### Sense of Coherence 13-Item Scale

The Sense of Coherence 13-item scale (SOC-13) is a short version of Antonovsky’s Sense of Coherence Scale, which evaluates a person’s capacity to cope with stressors. The items are rated on a 7-point Likert scale, and ratings of all items are summed to generate a total score. Besides the total scale, the SOC-13 has three subscales: Meaningfulness (4 items), Comprehensibility (5 items), and Manageability (4 items). Higher scores indicate stronger SOC. Higher scores indicate stronger SOC. An example item from the Meaningfulness subscale is “How often do you have the feeling that there’s little meaning in the things you do in your daily life,” an example item from the Comprehensibility subscale is “Has it happened in the past that you were surprised by the behavior of people whom you thought you knew well?” and an example item from the Manageability subscale is “Has it happened that people whom you counted on disappointed you?” The SOC-13 has been shown to have sufficient validity and reliability (Antonovsky, 1993). The Chinese version of the SOC-13 has acceptable internal consistency reliability, with a Cronbach’s alpha coefficient of 0.76 for the total score (Ding et al., 2012).

### Statistical Analysis

Data analyses were conducted using SPSS version 21. Descriptive statistics (means, standard deviations, ranges, and frequencies) were calculated to profile the sociodemographic and disease-related characteristics of the sample. Harman’s single factor test was performed to examine the existence of common method bias, i.e., such bias was considered to exist if the 1st principal component explained more than 50% of the variance. Differences in SOC and HRQoL between subgroups of patients were tested using two-sample *t*-tests or one-way ANOVAs. Associations between scale scores were examined using Pearson’s correlation coefficients. The significance level was set at *p* < 0.05 (two-tailed). *Post hoc* statistical power was calculated based on the actual effect size, α error probability (0.05) and actual sample sizes using G^∗^Power software, version 3.1.9 (Faul et al., 2007).

## Results

### Sociodemographic and Clinical Information

The sociodemographic characteristics of the patients are presented in Table 1. The sample consisted of 162 patients (89 males and 73 females; mean age: 59.70 years, *SD* = 10.95, range: 27–83 years). Among the patients, 81 finished elementary or secondary school education, 47 completed high school or college education, and 22 received undergraduate or graduate education. Most (93.2%) patients were married, 83.3% had medical insurance, 92.6% did not report any religious affiliation, and 16.0% were not aware of the situation of BM. The mean time interval between diagnosis of primary cancer and brain metastasis was 22.66 months (*SD* = 36.82, range: 0–262 months). The average number of BM was 2.49 (*SD* = 3.07, range: 0–20). Forty-one (25.3%) patients had extracranial metastatic lesions. The patients’ functional impairment, as indicated by the Karnofsky Performance Scale (KPS) score (Karnofsky et al., 1948), which was rated by a nurse based on the medical record and an interview with patient or family, ranged from 30 (unable to care for self, very sick, hospital admission necessary) to 100 (no special care needed, no health complaints, able to work). The mean KPS score was 73.21 (*SD* = 20.02), which corresponded to being able to care for oneself, but unable to conduct normal activities or engage in active work.

**TABLE 1:** Sociodemographic and clinical characteristics of the patient sample (*n* = 162).

**Characteristic**	***n***	**%**
**Gender**		
Male	89	54.9
Female	73	45.1
**Marital status**		
Married	151	93.2
Single or divorced	11	6.8
**Education level**		
Primary/secondary school	81	50
High school and college	44	27.2
University and above	37	22.8
**Medical insurance**		
Self-paid	27	16.7
Medical insurance supported	135	83.3
**Religious affiliation**		
Yes	12	7.4
No	150	92.6
**Awareness of the state of illness**		
Yes	136	84.0
No	26	16.0
**Primary cancer**		
Lung cancer	120	74.1
Breast cancer	16	9.9
Gastrointestinal cancer	12	7.4
Others (i.e., genitourinary cancer)	14	8.6
**Extracranial metastatic lesions**		
Yes	41	25.3
No	121	74.7
**Treatment of the primary cancer^a^**		
Surgery	67	41.3
Chemotherapy	75	46.3
Targeted therapy	65	40.1

**^*a*^The total sums to more than 100% because patients may have received more than one type of treatment.**

### Health-Related Quality of Life in Patients With BM

Scores on the QLQ-C30 subscales indicated specific aspects of the quality of life of patients with BM. The Cronbach’s alpha coefficient of the QLQ-C30 scores of the patients was 0.914, indicating high internal consistency reliability. Compared with a large normative sample of 15,386 healthy subjects (Nolte et al., 2019), the patients with BM in the present study provided lower scores for most functioning subscales of the QLQ (physical, role, cognitive, and social functioning, *ps* < 0.001, Table 2), except for the emotional functioning subscale (*p* = 0.491). The rating of general quality of life also decreased in patients with BM (*p* < 0.001, Table 2), and the patients provided higher scores for most of the symptom scales (fatigue, pain, nausea and vomiting, dyspnea, insomnia, appetite loss, and constipation) and financial difficulties (*ps* < 0.003, Table 2), except for the diarrhea symptom subscale (*p* = 0.244). The *post hoc* statistical power values were larger than 0.996 for the significant differences.

**TABLE 2:** Comparison of QLQ-C30 scores in patients with BM (*n* = 162) and a normative sample of healthy controls (*n* = 15386).

	**Patients with BM**		**Normative sample**		***t***	***p*-value**	**Hedges’ *g***
				
	***M***	***SD***	***M***	***SD***			
PF	**61.04**	**34.35**	**85.1**	**18.9**	**−8.902**	**< 0.001**	**1.258**
RF	**56.58**	**38.13**	**84.3**	**24.6**	**−9.231**	**< 0.001**	**1.119**
EF	72.84	24.97	74.2	24.7	−0.690	0.491	0.055
CF	**76.99**	**26.85**	**84.8**	**21.3**	**−3.688**	**< 0.001**	**0.366**
SF	**56.38**	**35.69**	**86.2**	**24.1**	**−10.610**	**< 0.001**	**1.230**
FA	**44.10**	**29.86**	**29.5**	**25.5**	**6.199**	**< 0.001**	**0.571**
NV	**16.67**	**27.05**	**5.9**	**16**	**5.057**	**< 0.001**	**0.667**
PA	**34.98**	**33.24**	**23.5**	**27.1**	**4.380**	**< 0.001**	**0.423**
DY	**27.98**	**31.29**	**15.9**	**24.6**	**4.900**	**< 0.001**	**0.489**
IN	**34.98**	**34.61**	**26.6**	**30.3**	**3.069**	**0.003**	**0.423**
AP	**31.07**	**33.87**	**10.0**	**21.6**	**7.900**	**< 0.001**	**0.968**
CO	**26.34**	**33.11**	**12.5**	**23.3**	**5.305**	**< 0.001**	**0.591**
DI	11.59	22.68	9.5	20.9	1.170	0.244	0.100
FI	**47.32**	**36.35**	**10.6**	**23.6**	**6.562**	**< 0.001**	**1.545**
QL	**52.73**	**25.42**	**66.1**	**21.7**	**−6.672**	**< 0.001**	**0.615**

**Significant statistics (p < 0.05, two-tailed) are in bold. The normative sample contained 15,386 European healthy controls (Nolte et al., 2019). PF, physical functioning; RF, role functioning; EF, emotional functioning; CF, cognitive functioning; SF, social functioning; FA, fatigue; NV, nausea and vomiting; PA, pain; DY, dyspnea; IN, insomnia; AP, appetite loss; CO, constipation; DI, diarrhea; FI, financial difficulties; QoL, quality of life.**

### Correlation Between Sense of Coherence and Health-Related Quality of Life

For all patients, the mean SOC total score was 66.02 (*SD* = 11.66). The mean subscale scores were 25.30 (*SD* = 5.64) for Comprehensibility, 20.04 (*SD* = 4.21) for Meaningfulness, and 20.68 (*SD* = 4.30) for Manageability. The Cronbach’s alpha coefficient of the SOC scores of the patients was 0.833, indicating high internal consistency reliability. The SOC total and subscale scores were positively correlated with the global health status according to the QLQ-C30 and the four subscales measuring physical, role, emotional, and social functioning (Table 3). Additionally, SOC Manageability scores were correlated with cognitive functioning scores of the QLQ-C30, but not with SOC total, comprehensibility, and meaningfulness scores (Table 3). The SOC total score and subscale scores were negatively correlated with symptom scores (fatigue, pain, nausea and vomiting, dyspnea, insomnia, appetite loss, and constipation) and financial difficulties of the QLQ-C30, but not with the symptom score for diarrhea (Table 3). *Post hoc* statistical power values were larger than 0.589 for the significant correlations. All subscale scores of SOC and HRQoL were included in Harman’s single factor test. The result indicated that the 1st principal component explained 39.00% of the variance, suggesting that no common method bias existed in our data.

**TABLE 3:** Correlations between SOC scores and HRQoL scores in patients with BM (*n* = 162).

			**SOC**			
			**Total**	**Comprehensibility**	**Meaningfulness**	**Manageability**
QLQ-C30	Functioning	PF	***r* = 0.316**	***r* = 0.251**	***r* = 0.252**	***r* = 0.282**
			***p* < 0.001**	***p* = 0.001**	***p* = 0.001**	***p* < 0.001**
		RF	***r* = 0.361**	***r* = 0.299**	***r* = 0.255**	***r* = 0.341**
			***p* < 0.001**	***p* < 0.001**	***p* = 0.001**	***p* < 0.001**
		EF	***r* = 0.316**	***r* = 0.270**	***r* = 0.305**	***r* = 0.203**
			***p* < 0.001**	***p* < 0.001**	***p* < 0.001**	***p* = 0.010**
		CF	*r* = 0.152	*r* = 0.093	*r* = 0.100	***r* = 0.194**
			*p* = 0.054	*p* = 0.240	*p* = 0.206	***p* = 0.013**
		SF	***r* = 0.382**	***r* = 0.276**	***r* = 0.287**	***r* = 0.395**
			***p* < 0.001**	***p* < 0.001**	***p* < 0.001**	***p* < 0.001**

	Symptom	FA	***r* = −0.316**	***r* = −0.257**	***r* = −0.245**	***r* = −0.280**
			***p* < 0.001**	***p* = 0.001**	***p* = 0.002**	***p* < 0.001**
		NV	***r* = −0.205**	*r* = −0.152	***r* = −0.237**	*r* = −0.124
			***p* = 0.009**	*p* = 0.053	***p* = 0.002**	*p* = 0.117
		PA	***r* = −0.281**	***r* = −0.287**	***r* = −0.198**	***r* = −0.192**
			***p* < 0.001**	***p* < 0.001**	***p* = 0.012**	***p* = 0.014**
		DY	***r* = −0.224**	***r* = −0.191**	*r* = −0.108	***r* = −0.255**
			***p* = 0.004**	***p* = 0.015**	*p* = 0.171	***p* = 0.001**
		IN	***r* = −0.243**	***r* = −0.232**	***r* = −0.176**	***r* = −0.182**
			***p* = 0.002**	***p* = 0.003**	***p* = 0.025**	***p* = 0.020**
		AP	***r* = −0.254**	***r* = −0.258**	*r* = −0.137	***r* = −0.219**
			***p* = 0.001**	***p* = 0.001**	*p* = 0.082	***p* = 0.005**
		CO	*r* = −0.131	***r* = −0.171**	*r* = −0.064	*r* = −0.069
			*p* = 0.097	***p* = 0.030**	*p* = 0.422	*p* = 0.382
		DI	*r* = −0.035	*r* = −0.105	*r* = 0.062	*r* = −0.020
			*p* = 0.659	*p* = 0.185	*p* = 0.431	*p* = 0.798

	FI		***r* = −0.262**	***r* = −0.201**	***r* = −0.243**	***r* = −0.207**
			***p* = 0.001**	***p* = 0.010**	***p* = 0.002**	***p* = 0.008**

	Global QoL		***r* = 0.291**	***r* = 0.266**	***r* = 0.201**	***r* = 0.246**
			***p* < 0.001**	***p* = 0.001**	***p* = 0.010**	***p* = 0.002**

**Significant statistics (p < 0.05, two-tailed) are in bold. SOC, sense of coherence; PF, physical functioning; RF, role functioning; EF, emotional functioning; CF, cognitive functioning; SF, social functioning; FA, fatigue; NV, nausea and vomiting; PA, pain; DY, dyspnea; IN, insomnia; AP, appetite loss; CO, constipation; DI, diarrhea; FI, financial difficulties; QoL, quality of life.**

To examine whether SOC was the key variable associated with HRQoL, and whether there exist some other variables closely related to HRQoL, an analysis was conducted to test the association between the sociodemographic/clinical/psychological variables and QLQ-C30 scores (Table 4). The results indicated that SOC scores were significantly associated with QLQ-C30 total scores (*r* = 0.379, *p* < 0.001), while the other variables, including gender, age, education level, marital status, possession of medical insurance, possession of religious belief, type of primary cancer, existence of extracranial metastatic lesions, interval between diagnosis of primary cancer and BM, and awareness of BM were not significantly associated with QLQ-C30 total scores (*ps* > 0.088).

**TABLE 4:** Association between sociodemographic and clinical characteristics and HRQoL in patients with BM.

			**QLQ-C30 total score**	
	**Subgroups**	***n***	**Mean (*SD*)**	**Statistics**
Gender	Male	89	70.70 (19.48)	*t*(160) = 1.179; *p* = 0.240
	Female	73	66.78 (22.78)	

Education level	Primary/Secondary school	81	68.96 (20.78)	*F*(2, 159) = 0.143; *p* = 0.867
	High school/College	44	67.75 (19.10)	
	University and above	37	70.27 (24.16)	

Marital status	Married	151	68.78 (20.99)	*t*(160) = −0.342; *p* = 0.733
	Single/Divorced	11	71.03 (22.87)	

Medical insurance	Insured	135	69.10 (21.23)	*t*(160) = 0.228; *p* = 0.820
	Uninsured	27	68.09 (20.51)	

Religious affiliation	Yes	12	68.46 (15.57)	*t*(160) = −0.081; *p* = 0.935
	No	150	68.97 (21.47)	

Type of primary cancer	Lung	120	70.64 (20.19)	*F*(3, 158) = 1.132; *p* = 0.338
	Breast	16	66.01 (26.49)	
	Gastrointestinal	12	64.41 (18.67)	
	Others	14	61.52 (23.15)	

Extracranial metastatic lesions	Yes	41	64.08 (21.41)	*t*(160) = −1.719; *p* = 0.088
	No	121	70.58 (20.76)	

Awareness of BM	Yes	136	68.74 (21.15)	*t*(160) = −0.261; *p* = 0.794
	No	26	69.92 (20.92)	

Age		162	/	*r* = −0.117; *p* = 0.139

SOC total		162	/	***r* = 0.379; *p* < 0.001**
				*r* = −0.118; *p* = 0.158
Interval between diagnosis of primary cancer and BM		146	/	

**Significant statistics (p < 0.05, two-tailed) are in bold.**

### Variables Associated With Sense of Coherence

To examine whether SOC was associated with sociodemographic or clinical characteristics in patients with BM, a comparison was performed among subgroups and correlations between variables were assessed (Table 5). There was a significant effect of religious affiliation (*t* = 2.122, *p* = 0.035, Hedges’ *g* = 0.635), whereby those who expressed religious affiliation (*n* = 12, *M* = 28.58, *SD* = 4.96) reported higher scores on the Comprehensibility subscale of the SOC than those who did not express religious affiliation (*n* = 150, *M* = 25.03, *SD* = 5.61). Compared with patients who were not aware of the BM, patients who knew the extent of their disease reported significantly lower SOC total scores (*t* = −2.721, *p* = 0.007, Hedges’ *g* = 0.582) and scores for the two subscale of Comprehensibility (*t* = −2.440, *p* = 0.016, Hedges’ *g* = 0.522) and Meaningfulness (*t* = −2.207, *p* = 0.029, Hedges’ *g* = 0.471). Comprehensibility scores of the SOC were also associated with the type of primary cancer [ANOVA: *F*(3, 158) = 3.510, *p* = 0.017, Cohen’s *f* = 0.403]; *post hoc* comparisons indicated that the patients with primary gastrointestinal cancer produced higher SOC Comprehensibility ratings than did patients with primary breast cancer and other types of primary cancer (esophageal, liver, kidney, genitourinary, and ovarian cancer). The other sociodemographic and clinical variables were not significantly associated with SOC scores, including gender, age, education level, marital status, possession of medical insurance, presence of extracranial metastatic lesions, and the amount of time between diagnosis of primary cancer and brain metastasis (*ps* > 0.072). The *post hoc* statistical power values were 0.557, 0.590, and 0.951, respectively, for religious belief, awareness of BM, and type of primary cancer.

**TABLE 5:** Association between sociodemographic and clinical characteristics and SOC in patients with BM.

			**SOC total**		**SOC comprehensibility**		**SOC meaningfulness**		**SOC manageability**	
						
	**Subgroups**	***n***	**Mean (*SD*)**	**Statistics**	**Mean (*SD*)**	**Statistics**	**Mean (*SD*)**	**Statistics**	**Mean (*SD)***	**Statistics**
Gender	Male	89	66.87 (11.90)	*t* (160) = 1.013; *p* = 0.313	25.69 (5.87)	*t* (160) = 0.970; *p* = 0.334	19.94 (4.64)	*t* (160) = −0.331; *p* = 0.741	21.24 (4.28)	*t* (160) = 1.813; *p* = 0.072
	Female	73	65.00 (11.35)		24.82 (5.34)		20.16 (3.65)		20.01 (4.26)	

Education level	Primary/Secondary school	81	65.46 (12.01)	*F* (2, 159) = 0.202; p = 0.817	25.01 (5.72)	*F* (2, 159) = 0.219; *p* = 0.803	19.90 (4.24)	*F* (2, 159) = 0.128; *p* = 0.880	20.54 (4.27)	*F* (2, 159) = 0.091; *p* = 0.913
	High school/College	44	66.41 (11.44)		25.48 (5.64)		20.07 (4.02)		20.86 (4.35)	
	University and above	37	66.81 (11.38)		25.70 (5.56)		20.32 (4.45)		20.78 (4.40)	

Marital status	Married	151	65.95 (11.48)	*t* (160) = −0.287; *p* = 0.775	25.23 (5.66)	*t* (160) = −0.538; *p* = 0.591	20.07 (3.96)	*t* (160) = 0.331; *p* = 0.741	20.65 (4.29)	*t* (160) = −0.396; *p* = 0.693
	Single/Divorced	11	67.00 (14.57)		26.18 (5.56)		19.64 (7.02)		21.18 (4.62)	

Medical insurance	Insured	135	66.18 (11.77)	*t* (160) = 0.373; *p* = 0.710	25.37 (5.49)	*t* (160) = 0.373; *p* = 0.710	20.16 (4.19)	*t* (160) = 0.759; *p* = 0.449	20.65 (4.41)	*t* (160) = −0.220; *p* = 0.826
	Uninsured	27	65.26 (11.28)		24.93 (6.45)		19.48 (4.34)		20.85 (3.74)	

Religious affiliation	Yes	12	71.17 (11.34)	*t* (160) = 1.595; *p* = 0.113	**28.58 (4.96)**	***t* (160) = 2.122; *p* = 0.035**	21.50 (3.78)	*t* (160) = 1.249; *p* = 0.214	21.08 (5.45)	*t* (160) = 0.333; *p* = 0.740
	No	150	65.61 (11.62)		**25.03 (5.62)**		19.93 (4.23)		20.65 (4.21)	

Type of primary cancer	Lung	120	66.18 (11.46)	*F* (3, 158) = 1.527; *p* = 0.210	**25.56 (5.29)**	***F* (3, 158) = 3.510; *p* = 0.017**	19.91 (4.28)	*F* (3, 158) = 0.573; *p* = 0.633	20.71 (4.19)	*F* (3, 158) = 1.991; *p* = 0.118
	Breast	16	61.38 (12.21)		**23.19 (4.78)**		19.50 (4.65)		18.69 (4.73)	
	Gastrointestinal	12	70.75 (9.74)		**28.67 (4.46)**		21.00 (3.91)		21.08 (3.78)	
	Others	14	66.00 (13.50)		**22.57 (8.35)**		21.00 (3.37)		22.43 (4.70)	

Extracranial metastatic lesions	Yes	41	63.85 (10.66)	*t* (160) = −1.383; *p* = 0.168	24.41 (5.49)	*t* (160) = −1.160; *p* = 0.248	19.51 (4.23)	*t* (160) = −0.935; *p* = 0.351	19.93 (4.17)	*t* (160) = −1.310; *p* = 0.192
	No	121	66.76 (11.93)		25.60 (5.68)		20.22 (4.20)		20.94 (4.33)	

Awareness of BM	Yes	136	**64.96 (11.80)**	***t* (160) = −2.721; *p* = 0.007**	**24.83 (5.73)**	***t* (160) = −2.440; *p* = 0.016**	**19.73 (4.31)**	***t* (160) = −2.207; *p* = 0.029**	20.40 (4.36)	*t* (160) = −1.969; *p* = 0.051
	No	26	**71.62 (9.21)**		**27.73 (4.49)**		**21.69 (3.21)**		22.19 (3.69)	

Age	/	162	/	*r* = 0.010; *p* = 0.901	/	*r* = 0.039; *p* = 0.622	/	*r* = −0.103; *p* = 0.193	/	*r* = 0.076; *p* = 0.336

Interval between diagnosis of primary cancer and BM	/	146	/	*r* = 0.057; *p* = 0.492	/	*r* = −0.002; *p* = 0.984	/	*r* = 0.145; *p* = 0.081	/	*r* = 0.012; *p* = 0.886

**Significant statistics (p < 0.05, two-tailed) are in bold.**

## Discussion

In the present study, we first examined HRQoL in patients with BM. We then determined that there was a significant positive relationship between SOC and HRQoL in the patients with BM, which has been observed for other types of chronic illness (Kristofferzon et al., 2018). In addition, we identified the variables associated with SOC in the patients with BM. SOC scores were associated with the patients’ awareness of their illness progression, religious status, and type of primary cancer.

Compared with the normative sample of a previous study (Nolte et al., 2019), the patients with BM in the present study presented decreased scores for most of the functioning subscales (physical, role, cognitive, and social functioning) and increased scores for most of the symptom subscales (fatigue, pain, nausea and vomiting, dyspnea, insomnia, appetite loss, and constipation), and financial difficulties. The overall quality of life ratings were also lower in our patients than in the normative sample. The emotional functioning scale and the diarrhea symptom scale scores were not significantly different from those of the normative sample. These observations, indicate that the HRQoL of patients with BM significantly decreases in terms of both functioning and symptom aspects.

Individuals with a strong SOC maintain and enhance health by coping with stressors effectively and flexibly (Aderhold et al., 2019). Previously, SOC has been found to predict quality of life in patients with Parkinson’s disease and stroke (Kristofferzon et al., 2018). A correlation between SOC and quality of life was also demonstrated in a study of patients with advanced cancer; high perceived level of control is associated with better adjustment to cancer, and high self-efficacy is associated with less emotional distress (Eriksson and Lindstrom, 2007). A longitudinal study indicated that SOC appears to be a resource that directly enhances quality of life directly (Hansson and Cederblad, 2004). In the present study, we for the first time validated the correlation between SOC and HRQoL in patients with BM, thereby highlighting the close relationship between SOC and HRQoL in patients with advanced-stage cancer. Only the diarrhea symptom scale of HRQoL was not significantly related to the level of SOC in the patients with BM, which is consistent with observations in other types of cancer (Gerasimčik-Pulko et al., 2009; Rohani et al., 2015); that is, there appears to be no general relationship between SOC and diarrhea symptoms in patients with cancer.

The experience of cancer is generally considered a low-control situation. In facing cancer, it is better for patients if they have a strong SOC, so they may perceive their life as meaningful despite the adversities they face (Ranchor et al., 2010). SOC has been considered a trait that remains relatively consistent over time, even after cancer diagnosis (Lindblad et al., 2016). In the present study, we examined whether associations exist between sociodemographic or clinical features and SOC. Among the patients we treat, it is not uncommon to observe family members protecting the patients from knowing the state of their illness and helping the patients make medical decisions. The results of this study indicate that not knowing about the presence of cancer metastases can help patients maintain higher SOC than if knowing about the metastases. Awareness of BM may cause patients to feel there are insufficient resources for them to cope with the disease progression, thereby engendering lower SOC. A strong positive correlation between SOC and religious belief has been found in individuals aged 65 and older (Stefanaki et al., 2014). Weaver and Flannelly (2004) reported that patients with cancer who rely on spiritual and religious beliefs to cope with their illness are more likely to use an active coping style in which they accept their illness and try to cope with it in a positive and meaningful way. In the present study, we confirmed that religious belief is related to SOC in patients with advanced cancer. We also found an association between SOC comprehensibility and the type of primary cancer, which suggests that SOC may be modulated by cancer types. This remains to be systematically evaluated in large samples in future studies.

Our study was subject to several limitations. First, the study only involved patients who were admitted before receiving treatment for BM. A long-term follow-up would be informative for the analysis of SOC and its impact on HRQoL alterations over time in patients with BM. Second, the study population was heterogeneous in that it included several types of primary tumors. Thus, this sample might only be representative of patients with metastatic brain cancer before stereotactic radiosurgery. In future studies, other treatments, such as surgery and whole-brain radiotherapy, should be included in addition to including more hospitals and cancer centers to gather additional information about the effect of treatments on patients’ SOC and HRQoL. The lowest statistical power value for the significant results was 0.557, which is not sufficiently high. This may have resulted from the relatively small number of patients within certain subgroups (e.g., patients with religious beliefs, patients unaware of their disease progress). Studies with larger sample sizes are needed to increase statistical power.

In summary, the results of the present study indicate that high SOC levels are associated with high HRQoL in most aspects, with the exception of diarrhea symptoms. The SOC of patients with BM was associated with having religious beliefs, awareness of the illness, and the type of primary cancer. Our study validated the close association between SOC and HRQoL in patients with BM, and identified the sociodemographic, clinical, and psychological characteristics associated with SOC. These results highlight the close relationship between SOC and HRQoL, and that SOC in patients with BM is associated with awareness of the illness and possessing religious beliefs.

## Data Availability Statement

The datasets generated for this study are available on request to the corresponding author.

## Ethics Statement

The studies involving human participants were reviewed and approved by the Ruijin Hospital Ethics Committee (2017), clinical ethics approval No. (28). The patients/participants provided their written informed consent to participate in this study.

## Author Contributions

XQ and B-WW: conceptualization, writing – review, and editing. XQ and NZ: data curation, analysis, and writing the original draft. XQ, S-JP, and PZ: investigation and resources. B-WW: supervision. All authors contributed to the article and approved the submitted version.

## Conflict of Interest

The authors declare that the research was conducted in the absence of any commercial or financial relationships that could be construed as a potential conflict of interest.
